# Genome Sequencing and Analysis of the Hypocrellin-Producing Fungus *Shiraia bambusicola* S4201

**DOI:** 10.3389/fmicb.2020.00643

**Published:** 2020-04-09

**Authors:** Ning Zhao, Dan Li, Bing-Jing Guo, Xin Tao, Xi Lin, Shu-Zhen Yan, Shuang-Lin Chen

**Affiliations:** College of Life Sciences, Nanjing Normal University, Nanjing, China

**Keywords:** *Shiraia bambusicola*, pathogenicity, phylogeny, secondary metabolites, biosynthesis

## Abstract

*Shiraia bambusicola* has long been used as a traditional Chinese medicine and its major medicinal active metabolite is hypocrellin, which exhibits outstanding antiviral and antitumor properties. Here we report the 32 Mb draft genome sequence of *S. bambusicola* S4201, encoding 11,332 predicted genes. The genome of *S. bambusicola* is enriched in carbohydrate-active enzymes (CAZy) and pathogenesis-related genes. The phylogenetic tree of *S. bambusicola* S4201 and nine other sequenced species was constructed and its taxonomic status was supported (*Pleosporales*, *Dothideomycetes*). The genome contains a rich set of secondary metabolite biosynthetic gene clusters, suggesting that strain S4201 has a remarkable capacity to produce secondary metabolites. Overexpression of the zinc finger transcription factor *zftf*, which is involved in hypocrellin A (HA) biosynthesis, increases HA production when compared with wild type. In addition, a new putative HA biosynthetic pathway is proposed. These results provide a framework to study the mechanisms of infection in bamboo and to understand the phylogenetic relationships of *S. bambusicola* S4201. At the same time, knowledge of the genome sequence may potentially solve the puzzle of HA biosynthesis and lead to the discovery of novel genes and secondary metabolites of importance in medicine and agriculture.

## Introduction

*Shiraia bambusicola*, belonging to the genus *Shiraia* and the phylum Ascomycota, is an important pathogenic and parasitic fungus that grows on the twigs of bamboos. It adversely affects the growth of bamboo and causes significant economic losses every year. *S. bambusicola* is widely distributed in many provinces of southern China and Japan (Ali and Olivo, 2002; Morakotkarn et al., 2007). The fruiting body of *S. bambusicola* has been used as a traditional Chinese medicine for the treatment of sciatica, pertussis, tracheitis, and rheumatic arthritis (Zhao et al., 2016). On the other hand, *S. bambusicola* is known as the main hyprocrellin-producing species.

**FIGURE 1:**
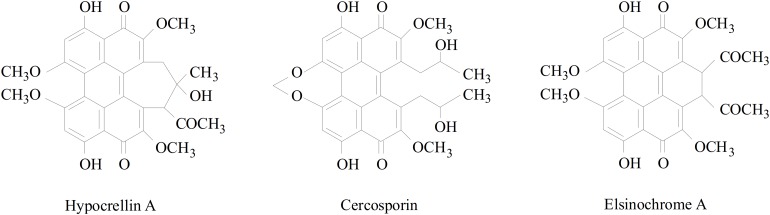
Structures of perylenequinones produced by plant pathogenic fungi.

The hyprocrellin family includes hypocrellin A (HA), hypocrellin B (HB), hypocrellin C (HC), and hypocrellin D (HD), which are isolated from the parasitic fungi *S. bambusicola* (Qi et al., 2014) and *Hypocrella bambusae* (Gu et al., 2005). Resembling many other natural perylenequinones (Figure 1), such as cercosporin and elsinochrome, the hypocrellin family is characterized by a helical chiral pentacyclic conjugated core structure combined with C7, C7′-substitutions, and possessing centrochiral stereochemistry (O’Brien et al., 2010). Because of its high potential pharmacological value, HA has attracted much attention. It has been shown to possess antibacterial, antiviral, antitumor, and anti-inflammatory bioactivities (Guo et al., 2017; Lin et al., 2017). As a photosensitizer, it can be activated under light irradiation and can produce reactive oxygen species (ROS), which can destroy DNA, proteins, and lipids (Robertson et al., 2009). Many researchers are studying the biological characteristics and fermentation production processes of *S. bambusicola*, as well as the applicability of photodynamic therapy of HA. Although many pharmacological effects of HA are being investigated, HA biosynthetic genes and pathways are still not well understood.

Fungi have specialized in using plant biomass as a carbon source by producing enzymes that degrade cell wall polysaccharides into metabolizable sugars for nutrition (Rytioja et al., 2014; Han et al., 2016). Carbohydrate-active enzymes (CAZy) are enzymes involved in the synthesis, metabolism, modification, and transport of carbohydrates. It is believed that the number of CAZymes in a fungal species correlates with its life strategies and the nutritional availability (Zhao et al., 2013). The Pathogen–Host Interaction database (PHI-base) contains expertly curated molecular and biological information on genes proven to affect the outcome of pathogen-host interactions. Cytochrome P450s (CYPs) are involved in the synthesis of toxins and pathogenesis (Nelson, 2011). The *S. bambusicola* S4201 genome data were entered in the databases mentioned above and we found some genes in *S. bambusicola* that may be involved in pathogenesis, providing a foundation for future studies of its lifestyle.

The taxonomic status of *S. bambusicola* has been reclassified several times over the past one hundred years. The genus *Shiraia* was initially classified under *Nectriaceae*, *Hypocreales*, and *Pyrenomycetes*, but was later transferred to the *Hypocreaceae* family based on the larger fleshy stroma. This taxonomic treatment was popular for several decades, but then *Shiraia* was transferred to the order *Pleosporales* based on its bitunicate, as opposed to unitunicate asci. According to the ninth edition of the fungal dictionary, *Shiraia* was classified under *Dothideales* incertae sedis (Kirk et al., 2008). Previous taxonomic classifications of *Shiraia* were mainly based on morphological characteristics of the ascostromata, ascus, ascospores, etc. However, several traditional morphological features are not class unique and DNA sequence comparisons are important to define the class. Recent studies sequencing the 18S rDNA and ITS-5.8S rDNA regions indicated that *Shiraia* should be classified under the order *Pleosporales* (Cheng et al., 2004). More recently, a new family, *Shiraiaceae*, has been proposed with *Shiraia* as the type genus. Its epitype was redescribed based on morphological characters and partial LSU-rDNA, EF, and RPB gene sequencing data, suggesting that it is a new family of *Pleosporales* (Liu et al., 2013). With the availability of more microbial genome sequencing data, researchers have begun to study phylogeny at the genomic level. However, the phylogeny of *S. bambusicola* has not been analyzed based on data at the genomic level. A phylogenetic analysis based on a small number of concatenated genes in any genome has a high probability of supporting conflicting topologies, while analysis of the entire data set of concatenated genes may provide a single, fully resolved species tree with maximum support (Rokas et al., 2003).

Fungal genomics and comparative analysis of different genomes are helpful in understanding phylogenetic and evolutionary relationships, gene function, lifestyle and strategies, biocontrol mechanisms, pathogenicity, and the development and utilization of secondary metabolites. Given the importance of these studies, we sequenced the *S. bambusicola* S4201 genome with Illumina HiSeq sequencing technology combined with PacBio single molecular long-read sequencing. A rich repertoire of CAZymes and pathogenesis-related genes were discovered. The taxonomic position of *S. bambusicola* was reconfirmed based on genomic data. A large set of secondary metabolic biosynthetic gene clusters and core genes were identified. Furthermore, we identified the HA gene cluster and proposed a new putative biosynthetic pathway. The information contained in this study could be useful for understanding the pathogenicity, taxonomic status, and diversity of the secondary metabolites of *S. bambusicola* S4201.

## Materials and Methods

### Strain, DNA, and RNA Isolation

*Shiraia bambusicola* S4201 was isolated in China from the fruiting bodies of *S. bambusicola* P. Henn, a strain which has shown excellent HA production and which has been used for sequencing (Zhao et al., 2016). The strain S4201 was incubated in potato dextrose broth (PDB, 200 g/L potato extract, 20 g/L glucose, pH 7.0) medium for 120 h at 28°C under agitation (150 r/min). The fermentation liquid was centrifuged at 3000 r/min for 5 min at room temperature and the supernatant was removed. The mycelium was washed with sterile water and frozen in liquid nitrogen. Genomic DNA was isolated using the cetyl trimethyl ammonium bromide (CTAB) method (Rogers and Bendich, 1994). The quality of the total DNA was confirmed by means of a Qubit Fluorometer, NanoDrop spectrophotometer, and by agarose gel electrophoresis before further processing.

### Genome Sequencing and Assembly

The *S. bambusicola* S4201 genome sequences included short reads from the Illumina library and long reads from a PacBio single-molecule library at the Beijing Genomics Institute (BGI) in Wuhan, China. A library with 410 bp inserts was constructed and sequenced using an Illumina Hiseq 4000 platform. To obtain clean data, 20 low-quality bases, 10% Ns, 15 bp overlap between adapter and duplications in the raw Illumina sequencing data were filtered. Total DNA (10 μg) was used to construct a 20 kb DNA library for sequencing with the PacBio platform. The PacBio data were also cleaned by removing adapter sequences, low-quality polymerase reads, and by discarding trimmed reads with lengths less than 1000 bp. The abundance of 15-mers was measured to obtain a preliminary estimate of the genome size, heterozygosity, and repetitive sequences information. Sequences were assembled using various assembly software and according to the following steps: (i) correcting the subreads to obtain the corrected reads (Proovread 2.12, -t 4 –coverage 60 –mode sr); (ii) assembling the corrected reads (Celera Assembler 8.3, doTrim_initialQualityBased = 1, doTrim_finalEvidenceBased = 1, doRemoveSpurReads = 1, doRemoveChimericReads = 1, -d properties -U); (iii) correcting the data using Illumina short reads (GATK v1.6-13, -cluster 2 -window 5 -stand_call_conf 50 -stand_emit_conf 10.0 -dcov 200 MQ0 ≥ 4) (Kim et al., 2014; Badouin et al., 2015; Faino et al., 2015; Sit et al., 2015; Tsuji et al., 2015).

### Gene Prediction and Annotation

The gene components were predicted by using Genewise (Birney et al., 2004), SNAP (Johnson et al., 2008), Genemark-ES (Ter-Hovhannisyan et al., 2008), and Augustus (Stanke et al., 2008) software. rRNA was aligned to an rRNA database or predicted by means of RNAmmer (Lagesen et al., 2007) software. tRNA and tRNA secondary structures were predicted with tRNA scan (Lowe and Eddy, 1997) and sRNA was matched with the Rfam (Gardner et al., 2009) database by using Infernal software. RepeatMasker and RepeatProteinMasker software were employed to examine the repeats present in *S. bambusicola* S4201. BuildXDFDatabase, RepeatModeler, and Repeatmasker were used for *de novo* detection. The tandem repeat sequences were identified using Tandem Repeat Finder (Benson, 1999) software. The protein-encoding genes were annotated through BLASTp searches in the Cluster of Orthologous Groups of proteins (COG) (Galperin et al., 2015; Makarova et al., 2015), Gene Ontology (GO) (Sherlock, 2009), Kyoto Encyclopedia of Genes and Genomes (KEGG) (Kanehisa et al., 2016), Non-Redundant (NR) Protein Database, SwissProt Databases and the best hits were filtered (*E-value* < 0.00001). CAZymes database (Levasseur et al., 2013) was used to identify proteins involved in carbohydrate metabolism. Pathogenicity and virulence-associated genes were predicted by means of the PHI database (Torto-Alalibo et al., 2009). CYPs were annotated using the Fungal CYP Database (Fischer et al., 2007).

### Comparative Genomics and Phylogenetic Tree

Since *S. bambusicola* S4201 is a single genus and species, we searched for several closely related species (*Leptosphaeria maculans*, *Parastagonospora nodorum*, *Paraphaeosphaeria sporulosa*, *Cercospora zeina*, *Stagonospora* sp. SRC1lsM3a, *Ascochyta rabiei*, *Diplodia corticola*, *Trichoderma citrinoviride*, *Neurospora crassa*) for evolutionary analysis. All of the related species genome sequences were downloaded from the National Center for Biotechnology Information (NCBI) database. To avoid the effects of alternative splicing, we chose the longest transcripts to represent the coding sequences. Gene families were defined by TreeFam methodology and then clustered with Hcluster_sg software (Li et al., 2006; Ruan et al., 2008). The orthologous groups and single-copy orthologs of these fungi were detected using the Perl script modified according to the programing code of Hacquard et al. (2016). Multiple sequence alignments of protein sequences were generated for each gene family using MUSCLE (Edgar, 2004), which converted the CDS alignments into protein alignments. A maximum likelihood phylogenetic tree was created using TreeBest^1^ with WAG amino acid substitution model. Synteny analysis and core-pan genes analysis were performed on four fungal species (*S. bambusicola*, *P. sporulosa*, *P. nodorum*, and *L. maculans*).

### Analysis of Core Genes and Gene Clusters Involved in Secondary Metabolism

The web-based prediction tool antibiotics and Secondary Metabolite Analysis Shell (antiSMASH) were used to predict secondary metabolite gene clusters and core genes (Blin et al., 2017). Based on previous RNA-Seq data from *S. bambusicola* S4201 (NCBI accession numbers: SRR2352154 and SRR2153022), we found the FPKM measurements of core genes which were commonly used to present gene expression levels. The *PKS* domain structures of different species were assigned according to the Conserved Domain Database (CDD) from the NCBI Search database and antiSMASH. The KS domain is the most conserved and is commonly used to infer the phylogenetic relationship between PKS genes. The predicted KS domains of *S. bambusicola* S4201 and other fungi were aligned by using ClustalW (Larkin et al., 2007) and the neighbor-joining tree was created with MEGA7, with 1000 bootstrap replicates (Kumar et al., 2016).

### Expression Vector Construction, Overexpression, and HPLC Profiling

The promoter *GPD*1, selection marker gene *ben*, promoter *GPD*2, and zinc finger transcription factor gene (*zftf*, as specialized to be involved in HA biosynthesis) were separately amplified from the S4201 genome and from pDHt-Ben using the primers OE-GPD-F1/OE-GPD-R1, OE-Ben-F1/OE-Ben-R1, OE-GPD-F2/OE-GPD-R2, and OE-zftf-F1/OE-zftf-R1. Then, the GPD1-Ben and GPD2-Zftf fragments were generated by overlapping PCR using PrimeSTAR^®^ Max DNA Polymerase (TaKaRa, Dalian, China) and primers OE-GPD-F1/OE-Ben-R1 and OE-GPD-F2/OE-zftf-R1. In addition, the GPD1-Ben fragment was cloned into the pEASY-Blunt Zero vector (TransGen Biotech) to generate the plasmid pGPD1-Ben. The GPD2-Zftf fragment was subcloned into the *Pme*I site of pGPD1-Ben, forming the plasmid pOE-*zftf*. *S. bambusicola* S4201 was grown in PDA at 28°C for 120 h. The spores were collected in sterile water, and the protoplasts were prepared by adding an enzyme mixture. The plasmids were introduced into *S. bambusicola* S4201 by the polyethylene glycol-calcium chloride (PEG-CaCl_2_) transformation method, according to published procedures (Jiang et al., 2014). After culturing for 120 h at 28°C, the transformants were identified by diagnostic PCR and the sequences were analyzed using the DNA of the mycelium. Primer sequences are shown in the supporting information section (Supplementary Table S1). Cultures of 1 × 10^5^ conidia per mL of S4201 and the overexpressing transformants were grown in PDB at 28°C under constant agitation at 150 r/min for 120 h. Next, the mycelia were used for detection of expression by qRT-PCR. The culture supernatant was extracted with ethyl acetate until it turned colorless. Then, the extracts were concentrated to dryness and the residue was dissolved in acetonitrile. The HA and elsinochrome A (EA) concentrations in the extracts were measured by high-performance liquid chromatography (HPLC) using standard reagents. HPLC was performed using an Agilent Technologies 1220 Infinity LC instrument. The HA and EA of *S. bambusicola* S4201 and the overexpression transformant were analyzed by HPLC at 30°C using a C18 column (5 μm, 4.6 × 250 mm) (SunFire^TM^, Waters, United States), with a flow rate of 1 mL/min and injection volume of 10 μL.

### Quantitative Real-Time PCR Analysis

The glyceraldehyde-3-phosphate dehydrogenase gene (*GAPDH*) was used as the internal reference. Total RNA was isolated using TRIzol reagent according to the instructions of the manufacturer (Vazyme, Nanjing, China). The RNA quality was assessed with 1.0% agarose gels and verified using NanoDrop 2000 (Thermo Fisher Scientific, United States). cDNA was synthesized using the HiScript II First Strand cDNA Synthesis Kit (+ gDNA wiper) (Vazyme, Nanjing, China). qRT-PCR was performed using a StepOnePlus^TM^ Real-Time PCR System (Applied Biosystems, United States) and AceQ qPCR SYBR Green Master Mix (Vazyme, Nanjing, China). The results were calculated using the 2^–ΔΔ*Ct*^ method. All the gene-specific primers are listed in Supplementary Table S2. All the experimental data were obtained from three biological replicates.

## Results

### *De novo* Genome Sequencing, Assembly, and Annotation

**FIGURE 2:**
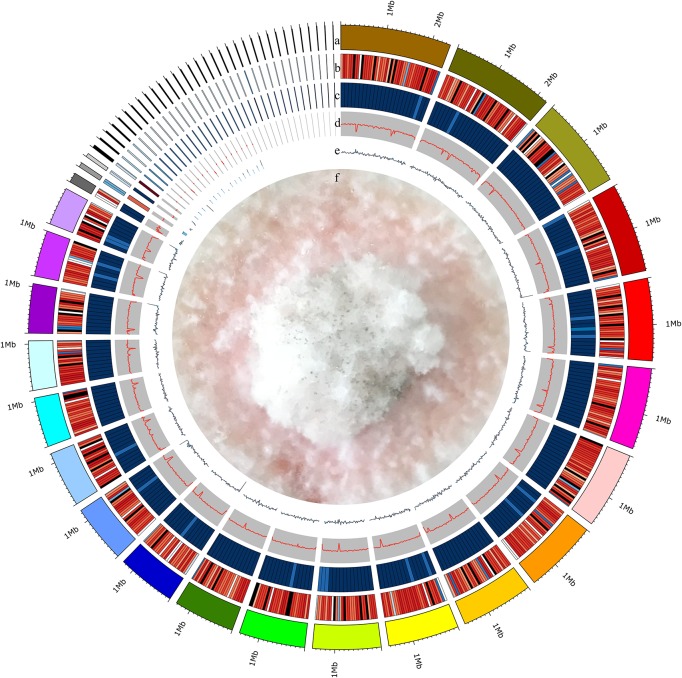
Circular representation of the *Shiraia bambusicola* genome. The following data are shown (from outer to inner): **(a)** Genome; sorted by length. **(b)** Gene density; gene number in 50,000 bp non-overlapping windows. **(c)** ncRNA density; ncRNA number in 100,000 bp non-overlapping windows. **(d)** GC; GC rate in 20,000 bp non-overlapping windows. **(e)** GC skew; GC skew in 20,000 bp non-overlapping windows. **(f)** The mycelia of *Shiraia bambusicola* S4201.

We isolated endophytic *S. bambusicola* S4201 fungi from the fruiting bodies of *S. bambusicola* P. Henn and demonstrated that it produced HA, based on HPLC analysis (Zhao et al., 2016). To gain insight into the pathogenicity, classification, and secondary metabolites of *S. bambusicola* S4201, we *de novo* sequenced and assembled its genome. By integrating Illumina HiSeq and PacBio single molecule long-read sequencing, we generated 2649 Mb of raw data and 4359 Mb of polymerase read bases. After removing the adapter sequence and low-quality reads, we obtained 2053 Mb of clean data and 4335 Mb of subread bases. The final *S. bambusicola* S4201 genome assembly included 32 Mb in 57 contigs, with a contig N50 of 1,565,644 bp (Figure 2). The genomic information was compared with that of *Shiraia* sp. slf14 (GenBank: AXZN00000000.1, no annotation, unpublished results) in the NCBI database (Table 1). Moreover, the gene length distributions and open reading frames of the genome were predicted based on the assembly sequences (Supplementary Figure S1 and Supplementary Table S3). Non-coding RNA (ncRNA) statistics are shown in Supplementary Table S4. The repeats, long terminal repeats (LTRs), long interspersed elements (LINE), and short interspersed elements (SINE) are shown in Supplementary Tables S5, S6. The genome sequence of *S. bambusicola* S4201 has been deposited at GenBank under accession number SRR8379567.

**TABLE 1:** Characteristics of the genome of *Shiraia bambusicola*.

Sample	*Shiraia bambusicola* S4201	*Shiraia* sp. slf14
Genome size (bp)	32,655,727	32,067,383
Contig	57	288
N50 (bp)	1,565,644	525,954
GC content	47.91	48
Chromosome number	57	0
Genome coverage	100×	55×
Gene number	11,332	–
ncRNA number	438	–
Repeat size (bp)	1,294,534	–
Annotation number	10,307 (90.95%)	–
Sequencing technology	Illumina HiSeq + PacBio	Illumina HiSeq

To conduct functional annotation of the S4201 gene model, we used the blast search function to enter the putative protein-coding sequences into the GO, KEGG, COG, Swiss-Prot, Trembl, GenBank NR, EggNOG (Huerta-Cepas et al., 2016), and TransportDB databases. There were 6358 (56.10%) annotated genes obtained for the three main GO categories of biological process, cellular component, and molecular function, including 48 sub-categories (Supplementary Figure S2). In total, 4368 (38.54%) genes were annotated and assigned to 45 different KEGG pathways (Supplementary Figure S3). “Carbohydrate metabolism” was the most enriched pathway, followed by “Amino acid metabolism” and “Translation.” In summary, 1318 (11.63%) genes were annotated into the COG database (Supplementary Figure S4). Among the 25 COG functional categories, the cluster for “General function prediction only” was the largest, followed by “Amino acid transport and metabolism” and “Carbohydrate transport and metabolism.” Only a few genes were assigned to the “Extracellular structures,” “Chromatin structure and dynamics,” and “RNA processing and modification” groups.

### Genes Involved in Pathogenicity

**FIGURE 3:**
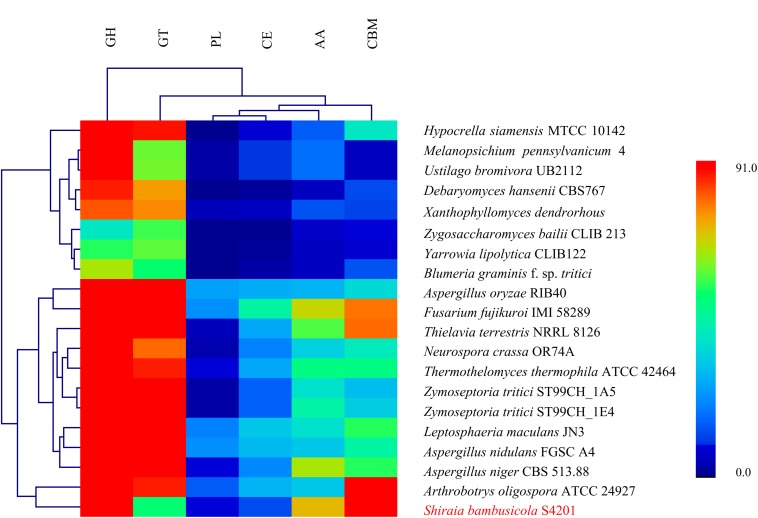
Hierarchical clustering of CAZyme in the genomes of *Shiraia bambusicola* and 19 other fungal species. The enzyme families are classified as follows: GH, glycoside hydrolases; GT, glycosyl transferases; PL, polysaccharide lyases; CE, carbohydrate esterases; AA, auxiliary activities; and CBM, carbohydrate-binding modules. The family numbers are based on the carbohydrate-active enzyme database. A color scale indicates the numbers of the different enzymes in each genome, from lowest (blue) to highest (red).

To produce a successful infection, phytopathogenic fungi often have to break down plant cell walls and penetrate into host tissues by means of CAZymes (Yong et al., 2014). The *S. bambusicola* S4201 genome encodes 414 putative CAZymes, including 166 glycoside hydrolases (GHs), 52 glycosyltransferases (GTs), nine polysaccharide lyases (PLs), 13 carbohydrate esterases (CEs), 72 auxiliary activities (AAs), and 102 carbohydrate-binding modules (CBMs). A heatmap based on the classification of CAZymes of 20 fungal species (*N. crassa* OR74A, *Aspergillus oryzae* RIB40, *Debaryomyces hansenii* CBS767, *Fusarium fujikuroi* IMI 58289, *Hypocrella siamensis* MTCC 10142, *L. maculans* JN3, *Melanopsichium pennsylvanicum* 4, *Thielavia terrestris* NRRL 8126, *Thermothelomyces thermophila* ATCC 42464, *Ustilago bromivora* UB2112, *Xanthophyllomyces dendrorhous*, *Yarrowia lipolytica* CLIB122, *Zygosaccharomyces bailii* CLIB 213, *Zymoseptoria tritici* ST99CH_1A5, *Z. tritici* ST99CH_1E4, *Arthrobotrys oligospora* ATCC 24927, *Aspergillus nidulans* FGSC A4, *Aspergillus niger* CBS 513.88, *Blumeria graminis* f. sp. *tritici*, *S. bambusicola* S4201) is shown in Figure 3. The AA coding ability of *S. bambusicola* S4201 was higher than that of other strains, and the CBM coding ability was second only to *A. oligospora* ATCC 24927. However, its GT coding ability was the weakest, lower than other selected strains. The proteins containing CAZy domains in the GH, PL, and CE groups may act as plant polysaccharide degradation (PDD) enzymes (Han et al., 2016). Regarding the PDD enzyme families, 99 sequences were predicted, distributed in 34 families (Supplementary Table S7). As a component of the plant cell wall and the intercellular spaces, pectin can provide nutrients to pathogenic fungi. *S. bambusicola* S4201 has many candidate pectinases and includes almost all pectinase families known in fungi, including PL1, PL3, PL4, GH78, GH88, GH95, GH105, and GH115. Moreover, some putative pathogenic factors were predicted, such as cutinase genes, which may be needed to catalyze plant cuticle degradation. In addition, proteins belonging to the GH6, GH7, GH39, and GH51 families, involved in the degradation of cellulose, hemi-cellulose, and pectin in plant cell walls, were also predicted. Eight hundred ninety genes in *S. bambusicola* S4201 were putatively involved in pathogenicity and virulence when analyzed with the PHI database, and this accounts for 7.85% of the total predicted genes (Supplementary Table S8). One thousand twenty-six putative P450s (9.05%) were predicted in *S. bambusicola* S4201 (Supplementary Table S9). In total, 74 major facilitator superfamily (MFS) transporters (Supplementary Table S10) and 46 ATP-Binding Cassette (ABC) transporters (Supplementary Table S11) were predicted.

### Comparative Genomics and Classification

**FIGURE 4:**
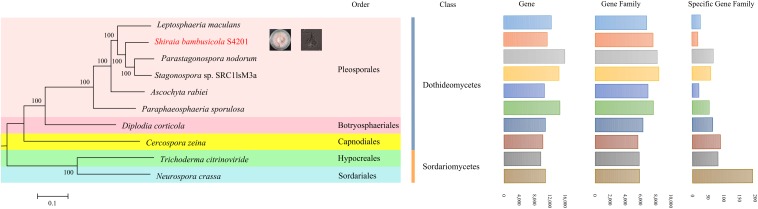
A maximum likelihood phylogenetic tree was inferred based on the highly conserved single copy orthologs of ten fungal genomes. The numbers on the branch points of the phylogenetic tree represent the bootstrap values (1000 replicates). Scale lengths represent the genetic distance. The 10 fungi are marked with different colors. The statistics for gene numbers, family numbers, and unique families in the 10 fungal species are shown.

In *S. bambusicola* S4201 and nine other selected fungal species (*L. maculans*, *P. nodorum*, *P. sporulosa*, *C. zeina*, *Stagonospora* sp. SRC1lsM3a, *A. rabiei*, *D. corticola*, *T. citrinoviride*, *N. crassa*), a total of 120,799 genes clustered into 10,851 gene families, including 3423 (31.55%) gene families shared by all species and 2105 (19.40%) that were single copy orthologs (Supplementary Table S12). The differences and quantities of ortholog groups among the fungi are shown in Supplementary Figure S5. The taxonomic status of *S. bambusicola* among other nine fungal species was evaluated. Based on highly conserved single copy orthologs, the phylogenetic tree (Figure 4) was constructed with the maximum likelihood method. The *S. bambusicola* S4201 genome was further characterized by comparative gene functional analyses with the following closely related species: *P. sporulosa*, *P. nodorum*, and *L. maculans*. Comparative analyses of the four available genomes revealed that *P. nodorum* had the largest number of genes. Synteny analysis showed that *S. bambusicola* S4201 and *P. nodorum* genome shared many large areas of synteny (Supplementary Figure S6) and they also share more homologous proteins. Comparative analysis of core and pan genes among the four species showed that there are 3480 genes classified as core genes, 35,395 genes classified as pan genes, and 5275 genes classified as dispensable genes (Supplementary Figure S7). The heatmap of dispensable genes in these species suggests that *S. bambusicola* S4201 has the fewest species specific genes (Supplementary Figure S8). It shows that *S. bambusicola* S4201 is more closely related to *P. nodorum* (*Phaeosphaeriaceae*, *Pleosporales*, *Dothideomycetes*) than to other species. The analysis revealed that *S. bambusicola* S4201 belongs among the species of *Pleosporales*, *Dothideomycetes*.

### Secondary Metabolites

Filamentous fungi can produce many secondary metabolites, such as bioactive compounds or mycotoxins, which have been used for the synthesis of pharmaceuticals. In fungi, the core genes, accessory genes, regulators, transporters, and other genes involved in the biosynthesis and modification of secondary metabolites are organized in clusters (Daub and Chung, 2009). Using the scaffolds as the query sequences for the antiSMASH 4.1.0 platform, 73 putative secondary biosynthetic gene clusters were predicted. In total, 15 polyketide synthases (PKS, 14 T1PKS, and one T3PKS), six non-ribosomal peptide synthases (NRPS), one linaridin, two terpenes, one indole, one cf_fatty_acid, 28 cf_putative, and two other gene clusters were identified in the *S. bambusicola* S4201 genome, distributed among 20 scaffolds. Cluster 22 shared 26% gene similarity with the patulin biosynthetic gene cluster, cluster 60 is likely to be an asperfuranone biosynthetic gene cluster (27% of genes show similarity) and cluster 72 acts as a cyclochlorotine biosynthetic gene cluster (25% of genes show similarity). These results indicate that these predicted gene clusters may produce the mentioned secondary metabolites in *S. bambusicola* S4201.

**FIGURE 5:**
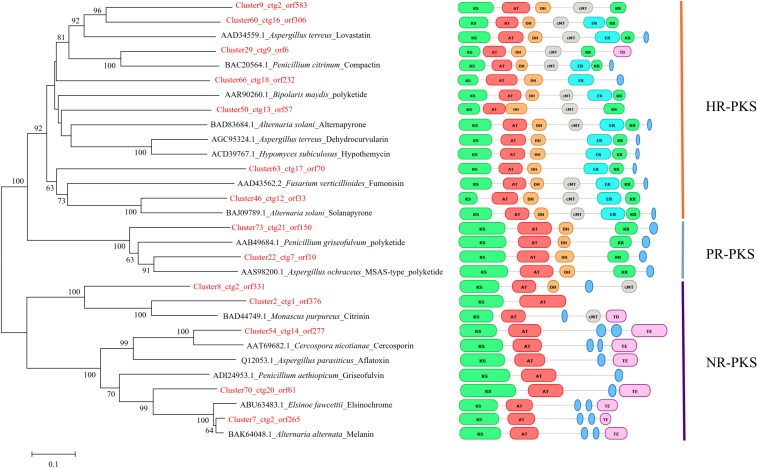
Phylogenetic analysis of the KS domain amino acid sequences of PKSs in *Shiraia bambusicola* S4201 and other fungal species. The numbers on the branch points of the phylogenetic tree represent the bootstrap values (1000 replicates). Bootstrap support values > 50% are shown above each branch. Scale lengths represent the genetic distance. Toxins or other polyketides are shown together with the fungal species. The *PKS* domain structures of different species are shown. The classification of their PKSs is also shown in the rightmost part of the figure.

To further understand the expression patterns of core biosynthetic genes involved in secondary metabolite biosynthesis, we estimated the expression of these genes in fragments per kb per million reads (FPKM) using previous RNA-seq data from *S. bambusicola* S4201 cultured for 82 h in PDB medium with Triton X-100 (Zhao et al., 2016). The secondary metabolite biosynthetic gene clusters are shown in Table 2. Phylogenetic analysis based on the KS domain amino acid sequences of PKSs in *S. bambusicola* S4201 and the products of known PKSs, was divided according to three main classes of PKSs, including highly reduced (HR) PKSs, partially reduced (PR) PKSs, and non-reduced (NR) PKSs (Figure 5). The reduced PKSs contain the reductive domains dehydratase (DH), enoyl reductase (ER), and keto-reductase (KR), while the NR-PKSs do not. ctg2_orf583, ctg16_orf306, ctg9_orf6, ctg18_orf232, ctg13_orf57, ctg17_orf70, and ctg12_orf33 were grouped with lovastatin and compactin synthase from *Aspergillus terreus* and *Penicillium citrinum* in the HR-PKS class. On the other hand, ctg21_orf150, ctg7_orf10, and ctg2_orf331 were nested in the PR-PKS clade. In addition, ctg1_orf376, ctg20_orf61, and ctg2_orf265 were distributed in the NR-PKS class, which include the polyketides cercosporin, aflatoxin, elsinochrome, and a putative polyketide involved in HA biosynthesis (ctg14_orf277).

**TABLE 2:** Secondary metabolite biosynthetic gene clusters in *Shiraia bambusicola* S4201.

Cluster ID	Core biosynthetic gene ID	Transcript ID	Type	FPKM	Domain structure
cluster2	ctg1_orf376	No hits found	T1PKS	–	KS-AT
cluster7	ctg2_orf265	comp5421_c0_ seq3	T1PKS	1.98	KS-AT-TE
cluster8	ctg2_orf331	No hits found	T1PKS	–	KS-AT-DH-cMT
cluster9	ctg2_orf583	comp12424_c0_seq1	T1PKS	5.09	KS-AT-DH-cMT-KR
cluster22	ctg7_orf10	comp5995_c0_ seq2	T1PKS	1.39	KS-AT-DH-KR
cluster29	ctg9_orf6	comp21554_c0_seq1	T1PKS	1.48	KS-AT-DH-cMT-KR-TD
cluster46	ctg12_orf33	comp22754_c0_seq1	T1PKS	0.90	KS-AT-DH-cMT-ER-KR
cluster50	ctg13_orf57	No hits found	T1PKS	–	AT-DH-cMT-KR
cluster54	ctg14_orf277	comp12566_c1_seq1	T1PKS	1,058.05	KS-AT-TE
cluster60	ctg16_orf306	comp5618_c0_ seq1	T1PKS	39.77	KS-AT-DH-cMT-ER-KR
cluster63	ctg17_orf70	comp19386_c0_seq1	T1PKS	1.27	KS-AT-DH-ER-KR
cluster66	ctg18_orf232	comp9563_c0_ seq1	T1PKS	1.62	AT-DH-ER
cluster70	ctg20_orf61	No hits found	T1PKS	–	KS-AT-TE
cluster73	ctg21_orf150	comp10038_c0_seq3	T1PKS	2.24	KS-AT-DH-KR
cluster64	ctg17_orf101	comp611_c0_ seq2	T3PKS	1.08	–
cluster4	ctg1_orf613	comp20525_c0_seq1	NRPS	0.00	A-TD
cluster11	ctg3_orf196	comp11842_c0_seq1	NRPS	20.45	C-A-C-A-E-C-A-C-A-E-C-C
cluster31	ctg9_orf134	comp70_c0_ seq1	NRPS	1.19	C
cluster36	ctg10_orf5	No hits found	NRPS	–	C-A-E-C-A-C-A-C-C-A-C-A-C-A-C
cluster39	ctg10_orf231	comp6571_c0_ seq2	NRPS	1.83	A-C-C
cluster72	ctg20_orf238	comp1287_c0_ seq1	NRPS	1.47	A
cluster3	ctg1_orf437	comp7504_c0_ seq1	Linaridin	110.33	–
cluster16	ctg5_orf244	comp12952_c0_seq1	Terpene	129.04	–
cluster65	ctg17_orf160	comp13731_c0_seq1	Terpene	50.50	–
cluster40	ctg11_orf2	No hits found	Indole	–	–
cluster42	ctg11_orf76	comp12067_c0_seq2	Other	4.12	A-NAD
cluster48	ctg12_orf304	comp8656_c0_ seq1	Other	2.76	A-TD-KR
cluster34	ctg9_orf346	comp12432_c0_seq5	Cf_fatty_acid	3.88	–

*T1PKS, Type I PKS (Polyketide synthase); T3PKS, Type III PKS; NRPS, non-ribosomal peptide synthetase cluster; Linaridin, linaridin cluster; Terpene, terpene; Indole, indole cluster; Other, cluster containing a secondary metabolite-related protein that does not fit into any other category; Cf_fatty_acid, possible fatty acid cluster.*

**FIGURE 6:**
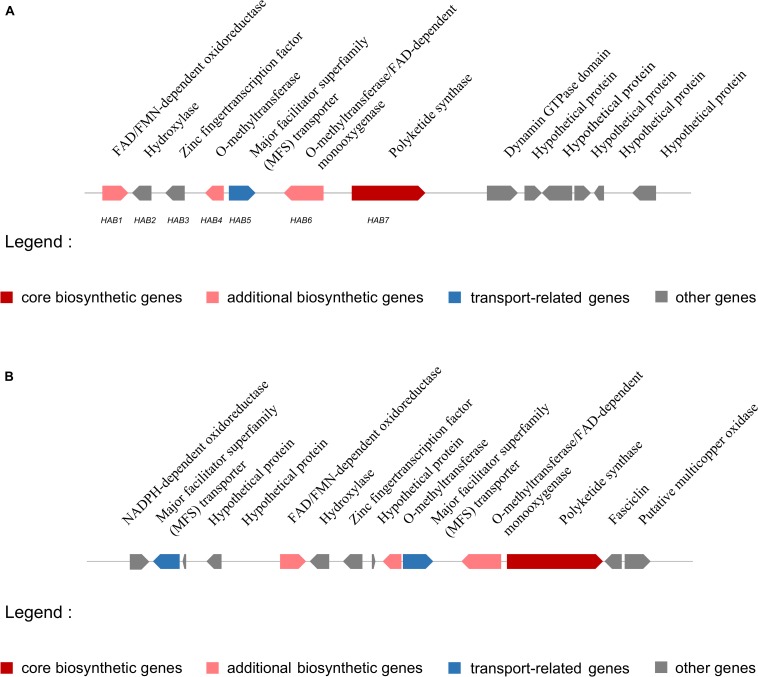
Putative hypocrellin A biosynthetic gene cluster in *Shiraia bambusicola* S4201 and *Shiraia* sp. slf14. **(A)** Putative hypocrellin A biosynthetic gene cluster in *Shiraia bambusicola* S4201. Cluster 54, with 13 genes, is located on scaffold 14 (1101201–1146665 nt) and was identified by using antiSMASH. Coding regions are indicated with arrows and are putatively involved in HA biosynthesis or have unknown functions. **(B)** Putative hypocrellin A biosynthetic gene cluster in *Shiraia* sp. slf14 based on the National Center for Biotechnology Information database (GenBank: KM434884.1).

The putative HA biosynthesis genes were classified into cluster 54, which was located on scaffold 14 (location: 1101201–1146665 nt) and included 13 genes (Figure 6). The genes in cluster 54 probably encode a FAD/FMN-dependent oxidoreductase (ctg14_orf271), a hydroxylase (ctg14_orf272), a zinc finger transcription factor (ctg14_orf273), an o-methyltransferase (ctg14_orf274), an MFS transporter (ctg14_orf275), an o-methyltransferase/FAD-dependent monooxygenase (ctg14_orf276), a polyketide synthase (ctg14_orf277), a dynamin GTPase domain (ctg14_orf278), and hypothetical proteins (ctg14_orf279–ctg14_orf283) (Supplementary Table S13).

### Overexpression of the Zinc Finger Transcription Factor *zftf*

A powerful approach to enhance the production of secondary metabolites is to overexpress Zn(II)Cys_6_-type pathway-specific transcription factors embedded in the corresponding gene clusters (Bennett, 2005; Bergmann et al., 2007). To determine the function of *zftf*, we cloned it into a plasmid, then transformed *S. bambusicola* S4201 with pOE-*zftf*, and the OE mutants were selected by diagnostic PCR amplification of the expression cassette. HPLC analysis with standard reagents (Supplementary Figure S9) showed that HA production by the OE mutant (206.75 mg/L) was higher than in wild type (82.60 mg/L). The expression levels of *zftf* in the mycelia were analyzed by quantitative real-time PCR (qRT-PCR), and the OE mutant showed more than threefold upregulation on average. These results show that the zinc finger transcription factor *zftf* plays an important regulatory role in HA production.

### Quantitative Real-Time PCR Analysis of Several Genes

**FIGURE 7:**
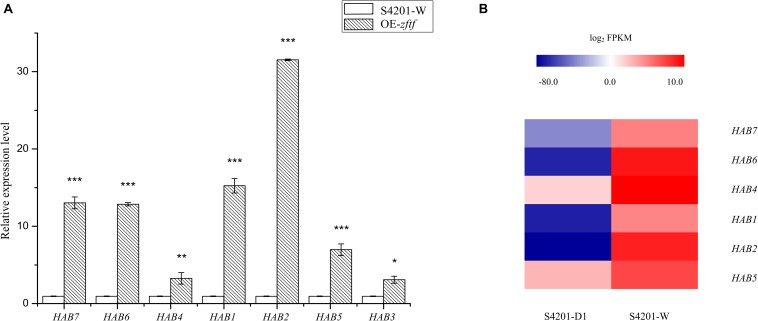
Expression of genes putatively involved in HA biosynthesis. **(A)** Quantitative RT-PCR validation. The relative mRNA expression levels were normalized with respect to the internal reference gene *GAPDH* and expression by S4201-W (control) was given an arbitrary value of 1. The abscissa shows the genes that encode polyketide synthase, o-methyltransferase/FAD-dependent monooxygenase, o-methyltransferase, FAD/FMN-dependent oxidoreductase, hydroxylase, major facilitator superfamily (MFS) transporter, and zinc finger transcription factor. *P < 0.05, **P < 0.01, ***P < 0.001 vs control group. **(B)** Heatmap of differentially expressed genes based on previous RNA-Seq data (Zhao et al., 2016). *Shiraia bambusicola* S4201-W shows abundant HA production, while the S4201-D1 mutant does not. A color scale indicates the expression levels of the different genes, from lowest (blue) to highest (red).

Seven genes (encoding polyketide synthase, o-methyltransferase/FAD-dependent monooxygenase, o-methyltransferase, FAD/FMN-dependent oxidoreductase, hydroxylase, MFS transporter, and zinc finger transcription factor) involved in HA biosynthesis were selected for qRT-PCR analysis to validate changes in gene expression. qRT-PCR analysis of total RNA extracted from wild type and the OE mutant grown in PDA showed that expression of these genes was upregulated in the OE mutant (Figure 7) indicating that the ZFTF transcriptional activator controls HA production by controlling gene transcript levels. Based on the previous RNA-Seq data (Zhao et al., 2016), the heatmap analysis of the expression of these genes in S4201-D1 and S4201-W was consistent with that in S4201-W and OE mutant. *S. bambusicola* S4201-W shows abundant HA production, while the S4201-D1 mutant does not. Thus, the HA biosynthesis core gene cluster is apparently coregulated through *zftf*, which encodes a Zn(II)Cys_6_ transcriptional activator, as expression of relevant genes was enhanced in the OE mutant.

## Discussion

*Shiraia bambusicola* is an important bamboo ascomycete pathogen. In this study, we sequenced, assembled, and analyzed the genome of this pathogen. The genome data are the initial step to understand the biology of *S. bambusicola*.

The chemical composition of bamboo fibers is mainly lignin, cellulose, and hemicellulose. Lignin accounts for the hardness and yellowness of bamboo fibers and is present at different concentrations in different layers of the cell wall (Khalil et al., 2012). In general, fungal pathogens possess pathogenic pathways which mediate cell wall degradation to enter host cells, interact with host cells, secrete toxins, and transport. During the infection stage, the polysaccharide content of the host cell wall is destroyed mainly through the action of CAZymes (Ospina-Giraldo et al., 2010), and these enzymes can also be used to utilize nutrients from the host cell. Analysis of CAZymes provides useful information about the lifestyles of fungi (Ohm et al., 2012). AAs include redox enzymes that act in conjunction with CAZymes. The AA group presently includes nine families of ligninolytic enzymes and six families of lytic polysaccharide monooxygenases (Levasseur et al., 2013). Among the 20 species analyzed, the AA content in *S. bambusicola* S4201 was the highest. The AAs aid in the hydrolysis of lignin, which are the main component of bamboo, providing a basis for *S. bambusicola*’s parasitism of bamboo. Apart from the catalytic modules, about 7% of CAZymes include CBMs, the most common non-catalytic modules linked to cell wall hydrolytic enzymes (Cantarel et al., 2009). They play an important role in the enzymatic hydrolysis of plant structures, in storage and in the degradation of other insoluble polysaccharides (Boraston et al., 2004). The higher content of CBMs in *S. bambusicola* S4201 may help it hydrolyze insoluble polysaccharides such as cellulase in bamboo, damaging cell walls and facilitating parasitism of this plant. Organisms with very large genomes that can synthesize complex cell walls or use glycosylation of small molecules to regulate biological activity have many GTs. By contrast, organisms that have experienced significant gene loss over the course of evolution and which have become obligate symbionts or obligate parasites appear to have little or no detectable GT genes (Lairson et al., 2008). Thus, it is of interest that the number of GTs in *S. bambusicola* S4201 was significantly less than in the other 19 species. As mentioned above, “Carbohydrate metabolism” was the most enriched pathway in KEGG pathways and lots of genes assigned to the “Carbohydrate transport and metabolism” in COG categories. Therefore, a large number of genes involved in carbohydrate transport and metabolism of *S. bambusicola* S4201 may be closely related to its pathogenicity. In the future, the pathogenicity analysis can be expanded as follows: first, by selecting at least four RNA samples from different infection stages or infection sites for transcriptome sequencing analysis. And second, by knocking out the selected pathogenicity-related genes to determine their relationship with *S. bambusicola* pathogenicity.

*Dothideomycetes* is regarded as the largest and most diverse class of Ascomycete fungi, and comprises 11 orders, 90 families, 1300 genera, and more than 19,000 known species. The species are taxonomically classified into three subclasses, 11 orders: *Capnodiales*, *Dothideales* and *Myriangiales* (*Dothideomycetidae*); *Hysteriales*, *Jahnulales*, *Mytilinidiales* and *Pleosporales* (*Pleosporomycetidae*); *Botryosphaeriales*, *Microthyriales*, *Patellariales*, and *Trypetheliales* (Incertae sedis). Among them, the most diverse fungal order in *Dothideomycetes* is *Pleosporales*, which represent roughly a quarter of all *Dothideomycetous* species (Kirk et al., 2008). In order to determine the classification of *S. bambusicola*, nine fungal species were used for phylogenetic construction using the complete genome sequencing data downloaded from the NCBI database. The results from the phylogenetic analysis were basically consistent with previous studies (Liu et al., 2013; Shen et al., 2015), which showed that *S. bambusicola* is located in the order *Pleosporales*. The inferred phylogeny indicated that *Stagonospora* sp. SRC1lsM3a and *P. nodorum* were most closely related to *S. bambusicola*; thus, they may share a common pathogenic fungal ancestor. Further studies are needed to clarify the evolutionary relationship between *S. bambusicola* and the proximal species. This is the first study supporting the taxonomic status of *S. bambusicola* at the genomic level. The analysis of synteny between four fungi in *Pleosporales* indicated that the *S. bambusicola* S4201 and *P. nodorum* genomes shared a high degree of synteny, followed by *P. sporulosa* and *L. maculans*. Although there is a degree of synteny among related species, we observed significant chromosome rearrangement. At the same time, *S. bambusicola* S4201 had the fewest species specific genes, suggesting that it was relatively conservative during its evolution.

In the secretion and transport stages, secondary metabolites act as virulence factors, and transport is also important for pathogenesis. These secondary metabolites, particularly mycotoxins in *S. bambusicola*, may be responsible for its adaptation to an exclusively parasitic life cycle. There are mainly two transport systems, ABC-transporter (Gao et al., 2019) and MFS-transporter (Wang et al., 2018). These analyses provide some insights into the role of CAZymes and secondary metabolites in the parasitic lifestyle of *S. bambusicola* S4201. Seventy-three secondary metabolite biosynthetic gene clusters were predicted in *S. bambusicola* S4201, 15 of which were polyketide synthase gene clusters. Numerous secondary metabolites have already been found (Liu et al., 2018), indicating there is an enormous potential for production of secondary metabolites by *S. bambusicola*. Based on the FPKM values in the previously reported RNA-Seq, we estimated the possible expression of the core genes in various secondary metabolite biosynthetic gene clusters, and the genes were generally believed to be expressed when the FPKM value ≥ 0.5. Nevertheless, fungal polyketides are complex to synthesize, due to the length of the polyketide chains, the degree of reduction of intermediates, cyclization and release of products, etc. Therefore, it is difficult to predict the final synthetic metabolic products based only on the DNA or amino acid sequences. First, uncertainty regarding repeated catalysis by polyketide synthase and unknown synthesis processes limit the ability to make predictions based on gene sequences alone. Second, many secondary metabolites are produced in very low amounts and are difficult to detect in wild fungi or are unexpressed in laboratory conditions, so the synthesis genes and products cannot be connected.

To study the phylogenetic relationship of PKSs in the genome of *S. bambusicola*, the structures of the synthetic products and the PKS reduction types, a phylogenetic tree was constructed based on the ketoacyl synthase domain (KS domain) amino acid sequence of the polyketide synthases of *S. bambusicola* and 16 other polyketide synthases. Because some of the predicted polyketide synthases of *S. bambusicola* were similar to the polyketide synthases producing known compounds, and clustered in the phylogenetic tree of the same branch, we speculated that *S. bambusicola* can produce the same compounds or similar structural analogs (Figure 5). We selected one of these compounds, EA, for HPLC detection. EA was detected when *S. bambusicola* S4201 was cultured in PDB for 120 h (Supplementary Figure S9). Since *S. bambusicola* can produce EA and HA, and their structures are similar (Figure 1), we believe that EA is also a type of photosensitizer and virulence factor that can cause bamboo disease.

Some HA biosynthetic genes in the cluster we predicted by antiSMASH differed from the ones in *Shiraia* sp. slf14 (GenBank: KM434884.1). Compared with *Shiraia* sp. slf14, the gene clusters we predicted lacked the NADPH-dependent oxidoreductase gene, MFS gene, fasciclin gene, putative multicopper oxidase gene, and three hypothetical proteins genes. In contrast, they included a dynamin GTPase domain, an unknown gene, and four hypothetical proteins genes. Comparison of putative HA biosynthetic gene cluster in *S. bambusicola* S4201 and *Shiraia* sp. slf14 is shown in Figure 6. In order to finally determine the function of these genes and whether they are involved in HA biosynthesis, further experiments such as gene knockout are still needed. The putative *S. bambusicola* S4201 PKS (cluster 54_ctg14_orf277) involved in HA biosynthesis are clustered in the same branch as the *Cercospora nicotianae* PKS, and their molecular structure and biosynthetic gene clusters are similar. Comparing the CTB and HA cluster genes, NADPH-dependent oxidoreductase (CTB6) and FAD-dependent monooxygenase (CTB7) appeared to be unique to the CTB cluster. Considering the function of these enzymes, it makes sense that they are absent in the HA cluster, given that these structural features are non-existent (Newman and Townsend, 2016). Future studies are needed to determine whether these genes participate in HA biosynthesis.

**FIGURE 8:**
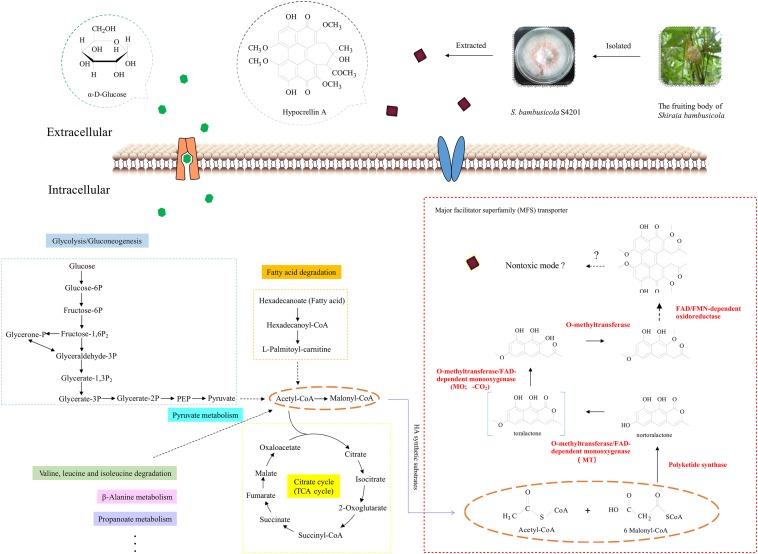
Schematic diagram of the central carbons of hypocrellin A showing its synthesis in *Shiraia bambusicola*. This flow chart simulates the process of HA synthesis. The highlighted and dotted lines on the left indicate the sources of HA substrates, and the genes marked with red on the right were upregulated in the transcriptome analysis we performed (Zhao et al., 2016). The proposed functions of the proteins encoded by the HA biosynthesis gene cluster are based on the biosynthesis studies of cercosporin (Newman and Townsend, 2016) and elsinochrome C (Chooi et al., 2017). The orange dotted lines represent the substrates for HA biosynthesis. The broken arrows represent putative steps, in which the enzymes involved are still unclear.

The Zn(II)Cys_6_ family of transcription factors can regulate various cellular processes in fungi (Thieme et al., 2018). When the *zftf* gene was overexpressed, expression of the genes in the putative HA cluster was increased, suggesting that the zinc finger transcription factor *zftf* plays a major role in the regulation of this pathway. The HPLC results also proved that overexpression of *zftf* increases HA production. A hypothetical HA biosynthetic pathway has been proposed based on the latest studies on cercosporin (Newman and Townsend, 2016) and elsinochrome C (Chooi et al., 2017), but it still needs to be validated with additional experiments. Figure 8 is a hypothetical flow diagram showing the biosynthesis of HA from substrates to its release by cells, in which the pathways are annotated based on the KEGG database. Due to the wide source of substrates, not all pathways are shown. Acetyl-CoA and malonyl-CoA are the precursors of polyketide biosynthesis, including HA. First, polyketide synthase synthesizes the common intermediate nor-toralactone. Second, o-methyltransferase/FAD-dependent monooxygenase methylates the intermediate at the OH-2 position and opens the pyrone ring, thereby introducing a C6-OH. O-methyltransferase/FAD-dependent monooxygenase is an unusual didomain enzyme with an o-methyltransferase domain and a flavin-dependent monooxygenase domain, and it has unique coupled activities. Third, o-methyltransferase methylates the nascent C6-OH. Fourth, FAD/FMN-dependent oxidoreductase catalyzes the dimerization, producing the perylenequinone carbon core. However, the downstream components and modification reactions in this pathway are still elusive. The HA in the fungal cell may be reduced to a non-toxic form that can minimize cell damage by oxidizing agents. Once synthesized, HA must be exported out of the cell by an MFS transporter. Once it is released, HA may spontaneously re-oxidize to its photoactive form. The identification of the HA pathway in *S. bambusicola* provides an opportunity to further study the biosynthesis of perylenequinones.

Current studies are mainly focused on the fermentation of *S. bambusicola* and the extraction of secondary metabolites. Future efforts should focus on identifying the genes involved in HA biosynthesis, the intermediate metabolites, and the synthesis pathways by gene knockout experiments and expression of heterologous proteins.

## Conclusion

*Shiraia bambusicola* is an important parasitic pathogen that grows on the twigs of bamboos and which has traditionally been considered to have anti-inflammatory and antiviral properties. We have *de novo* sequenced and assembled the genome of *S. bambusicola* S4201, which may serve as a basis for understanding its pathogenicity and exclusive parasitic lifestyle. *S. bambusicola* is capable of encoding a large and diverse set of CAZy, primary and secondary metabolism enzymes and transporters. The genome comparative study facilitated our understanding of the phylogeny of *S. bambusicola* and the evolutionary relationships between *S. bambusicola* and other sequenced species. Numerous secondary metabolite biosynthetic gene clusters were found. In addition, biosynthetic gene clusters for unknown products show the enormous potential that exists for drug discovery in *S. bambusicola*. Overexpression of the transcription factor *zftf* increased the production of HA by regulating the expression levels of other genes in the HA biosynthetic gene cluster. A new putative HA biosynthetic pathway was proposed. Our analyses provide some new insights into the pathogenicity, phylogeny, and secondary metabolites of *S. bambusicola*. In the future, this study will be an important resource for gene discovery and to increase HA production through further genetic manipulation.

## Data Availability Statement

The genome data datasets generated for this study can be found in the NCBI Repository with accession number SRR8379567. The RNA-Seq datasets from S. bambusicola S4201 generated for this study can be found in the NCBI Repository with accession numbers SRR2352154 and SRR2153022.

## Author Contributions

S-LC, S-ZY, and NZ conceived and designed the experiments. NZ, DL, and B-JG performed the experiments. NZ and XT analyzed the data. XL contributed reagents, materials, and analysis tools. NZ wrote the manuscript.

## Conflict of Interest

The authors declare that the research was conducted in the absence of any commercial or financial relationships that could be construed as a potential conflict of interest.

## Footnotes

1 http://treesoft.sourceforge.net/treebest.shtml
