# Supplementary material for: Genome Sequencing and Analysis of the Hypocrellin-Producing Fungus Shiraia bambusicola S4201
Source: Front Microbiol. 2020 Apr 9;11:643. doi: 10.3389/fmicb.2020.00643 (PMC7179677; doi:10.3389/fmicb.2020.00643)
Supplement: Supplementary file 1 [file Data_Sheet_1.DOCX]

***Supplementary Material***

## Supplementary Figures

**
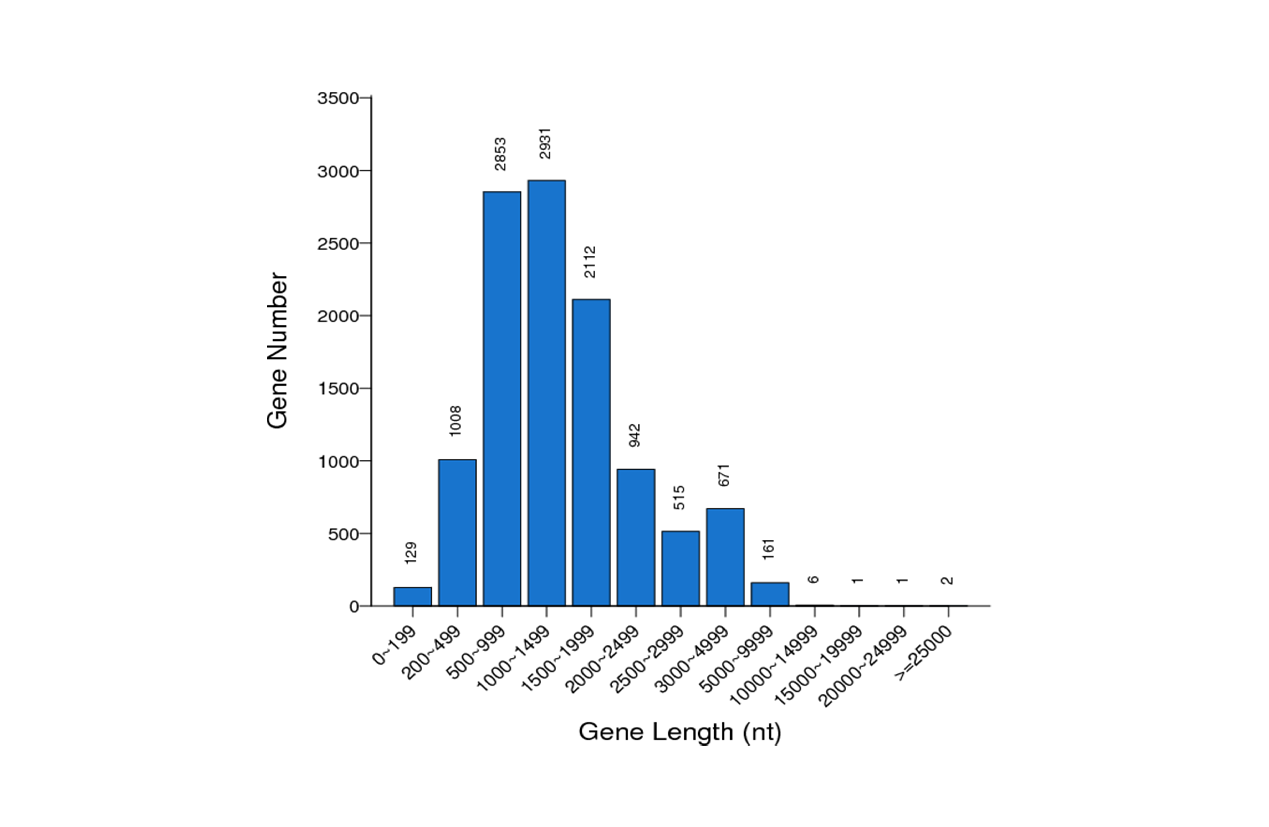
**

**Figure S1.** Gene length distributions in the *Shiraia bambusicola* S4201 genome.


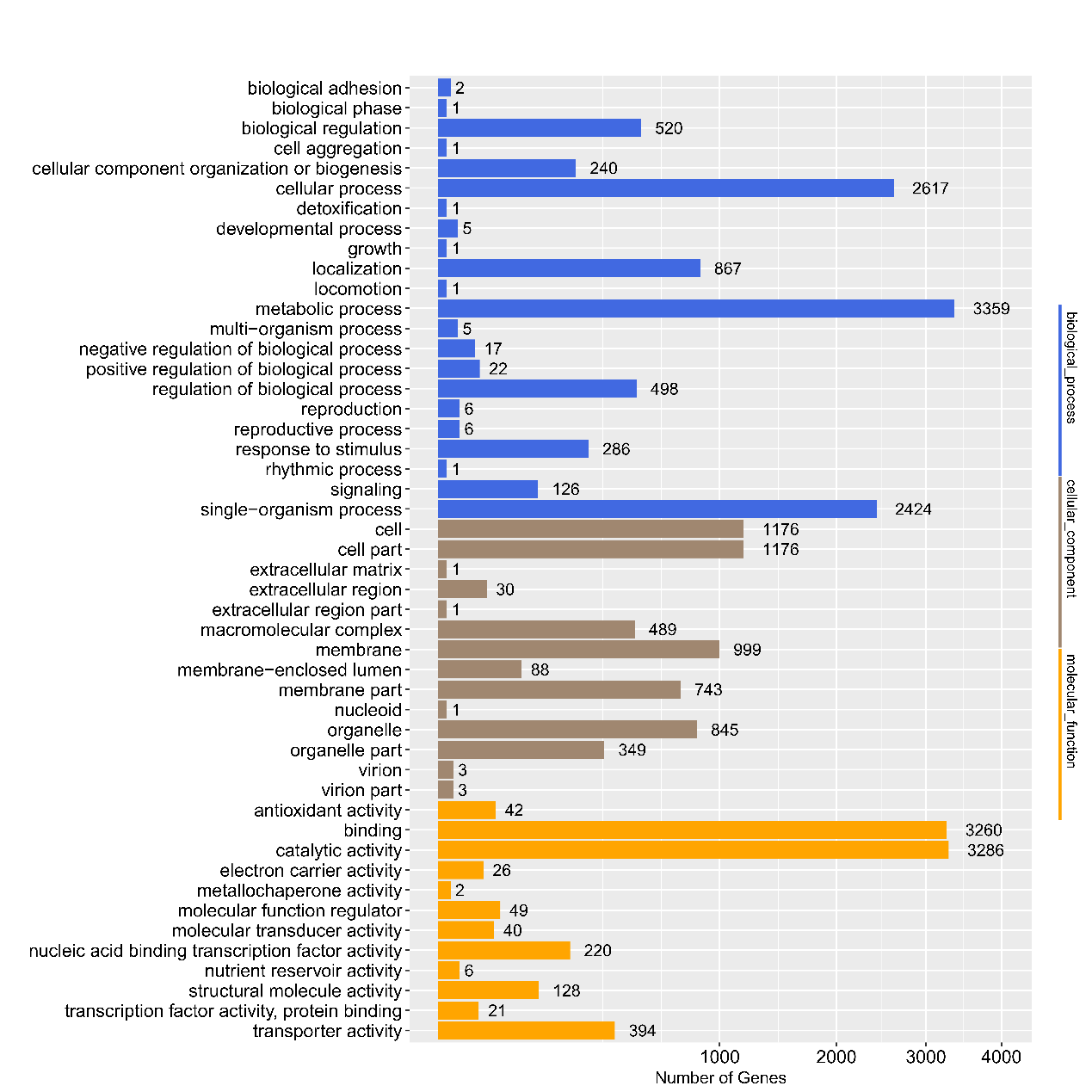


**Figure S2.** Gene Ontology classiﬁcation of the *Shiraia bambusicola* genome. A total of 6358 (56.10%) genes were assigned to the main GO categories [Biological processes (BP), Cellular components (CC) and molecular functions (MF)], including 48 sub-categories.


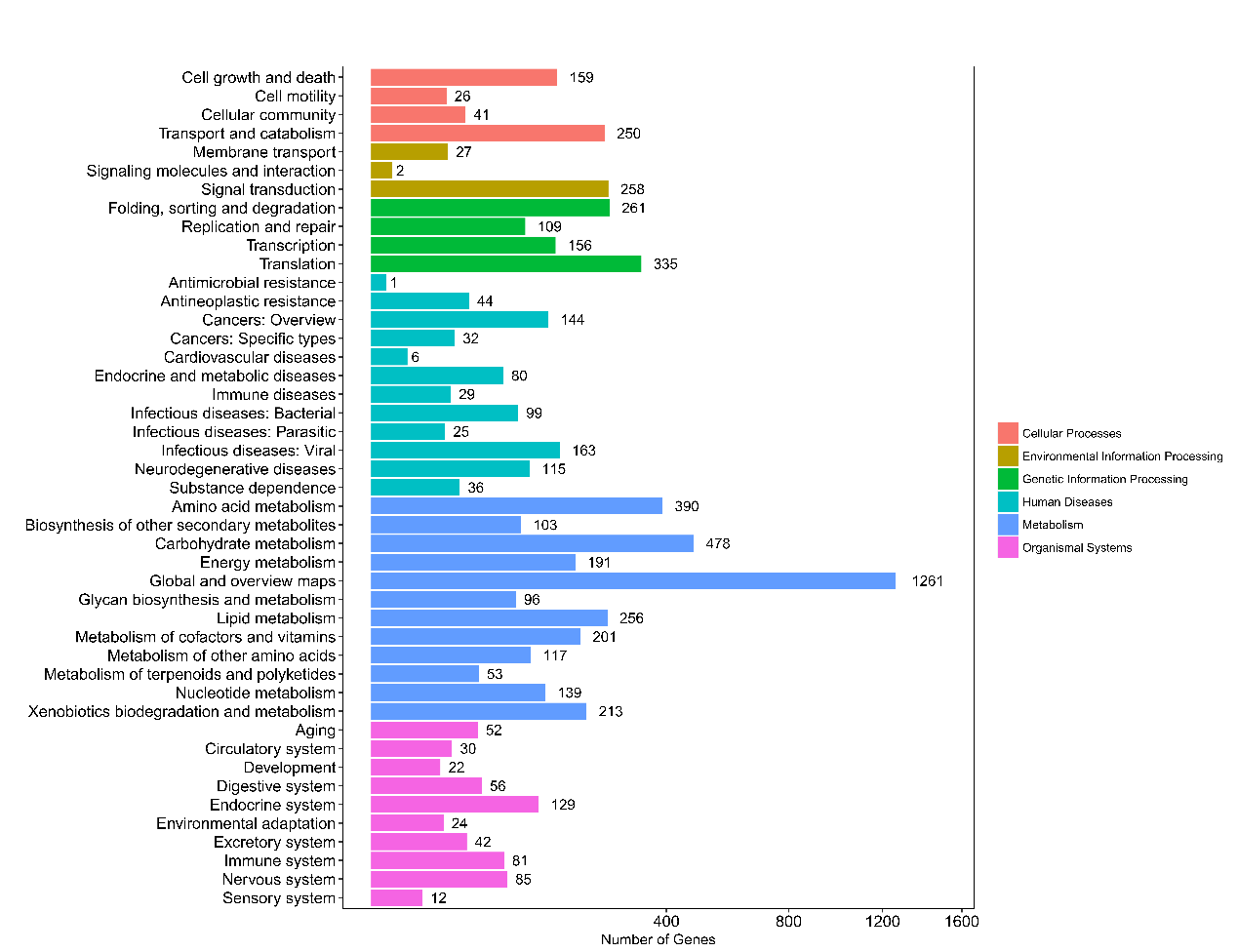


**Figure S3.** KEGG pathway classification of the *Shiraia bambusicola* genome. A total of 4368 (38.54%) genes were annotated and assigned to 45 different KEGG pathways.


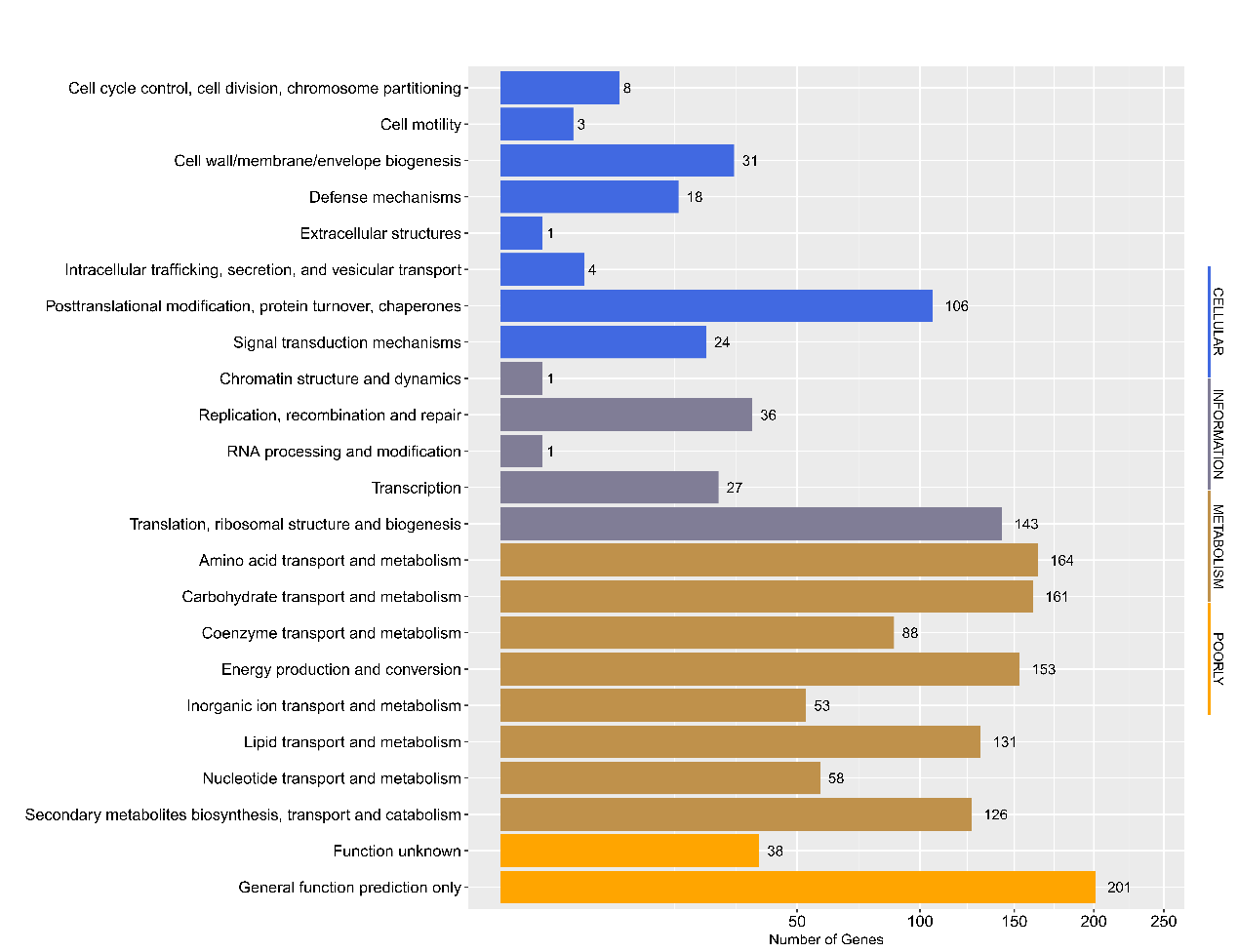


**Figure S4.** COG classification of the *Shiraia bambusicola* genome. A total of 1318 (11.63%) genes were clustered into 25 functional categories.


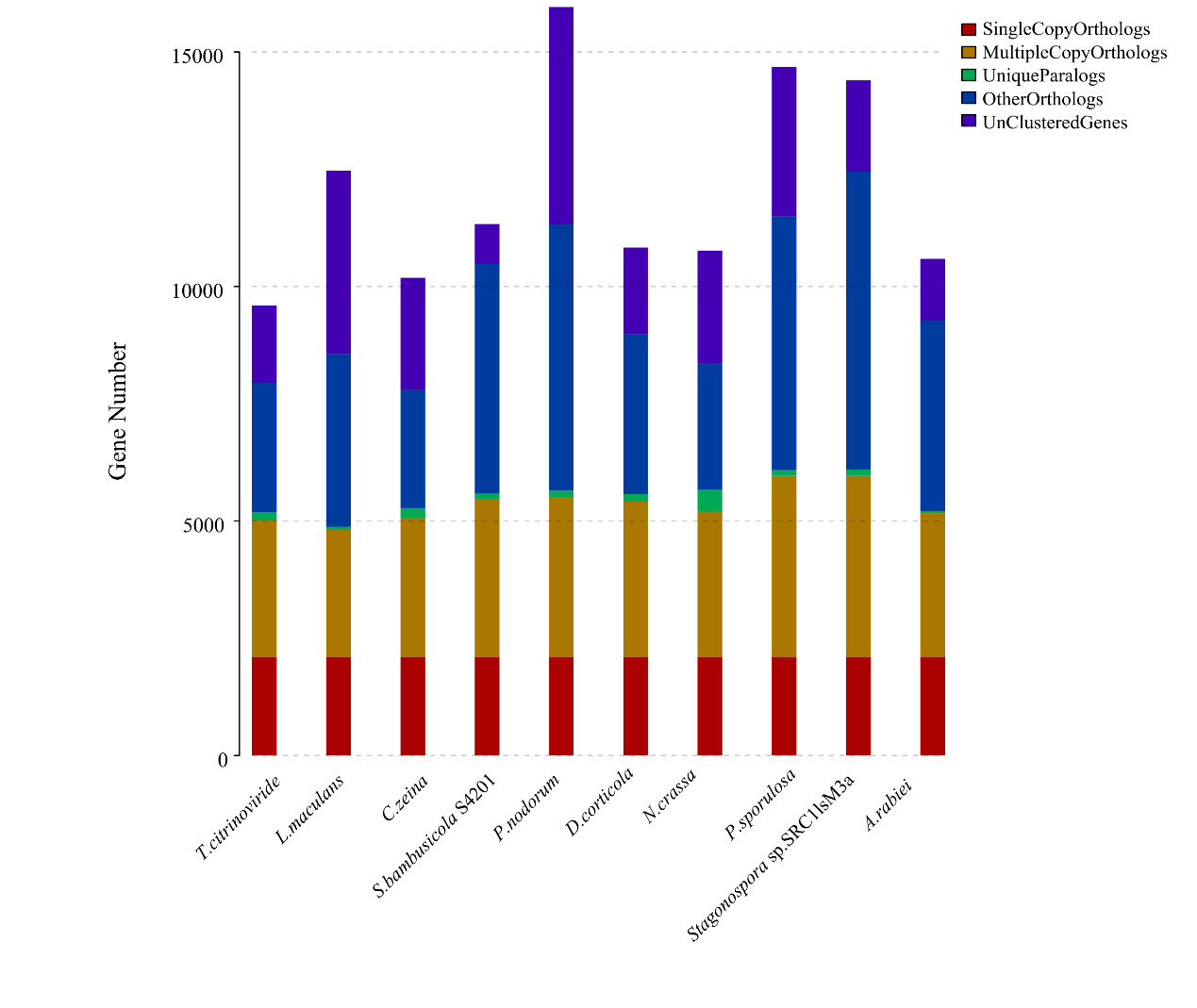


**Figure S5.** Classification of gene families in ten fungal species.


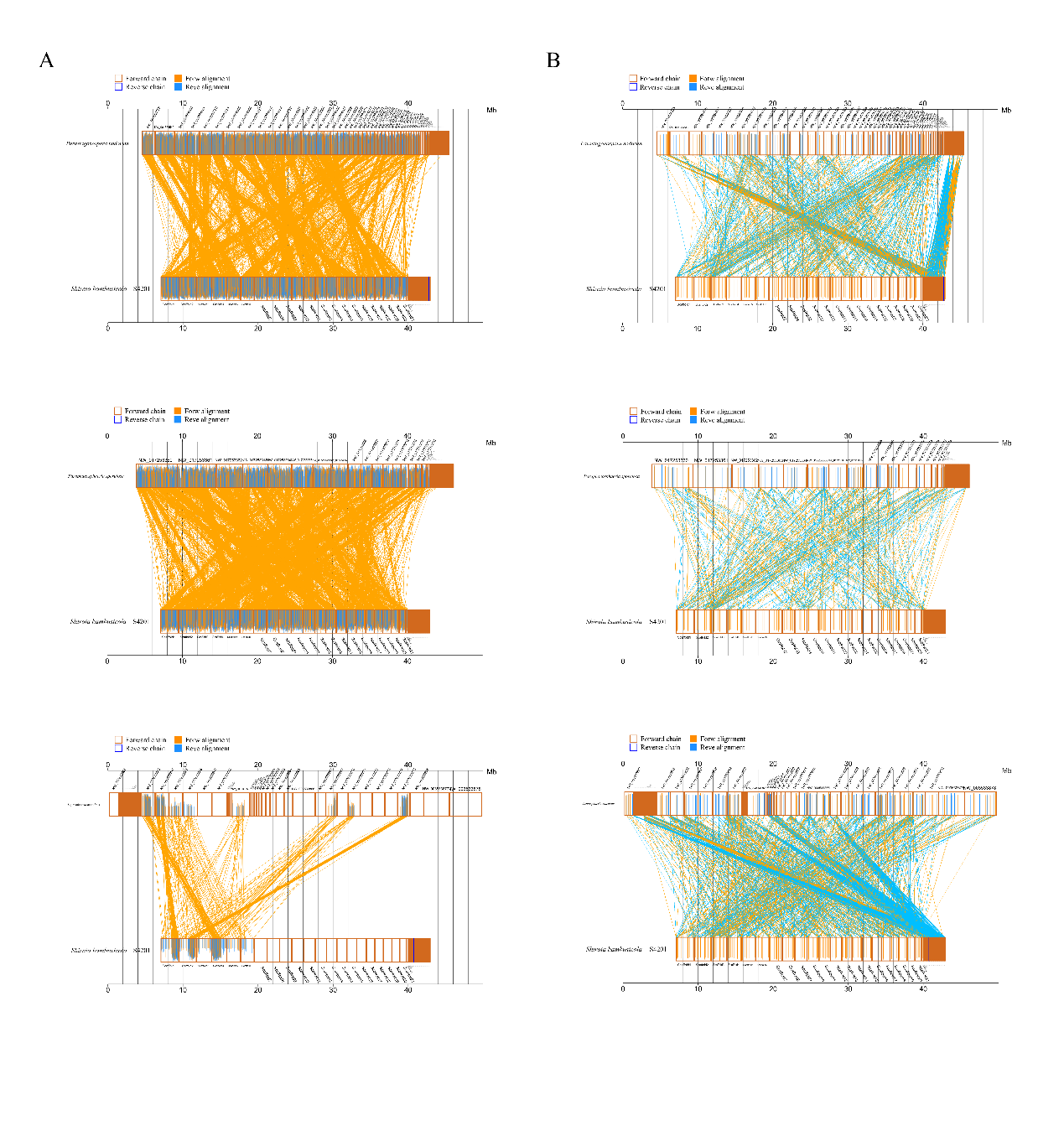


**Figure S6.** Synteny analysis of four genomes. (A) Synteny between *Paraphaeosphaeria sporulosa*, *Parastagonospora nodorum*, *Leptosphaeria maculans* and *Shiraia bambusicola* S4201 was analyzed at the amino acid level. (B) Synteny between *Paraphaeosphaeria sporulosa*, *Parastagonospora nodorum*, *Leptosphaeria maculans* and *Shiraia bambusicola* S4201 was analyzed at the nucleic acid level.


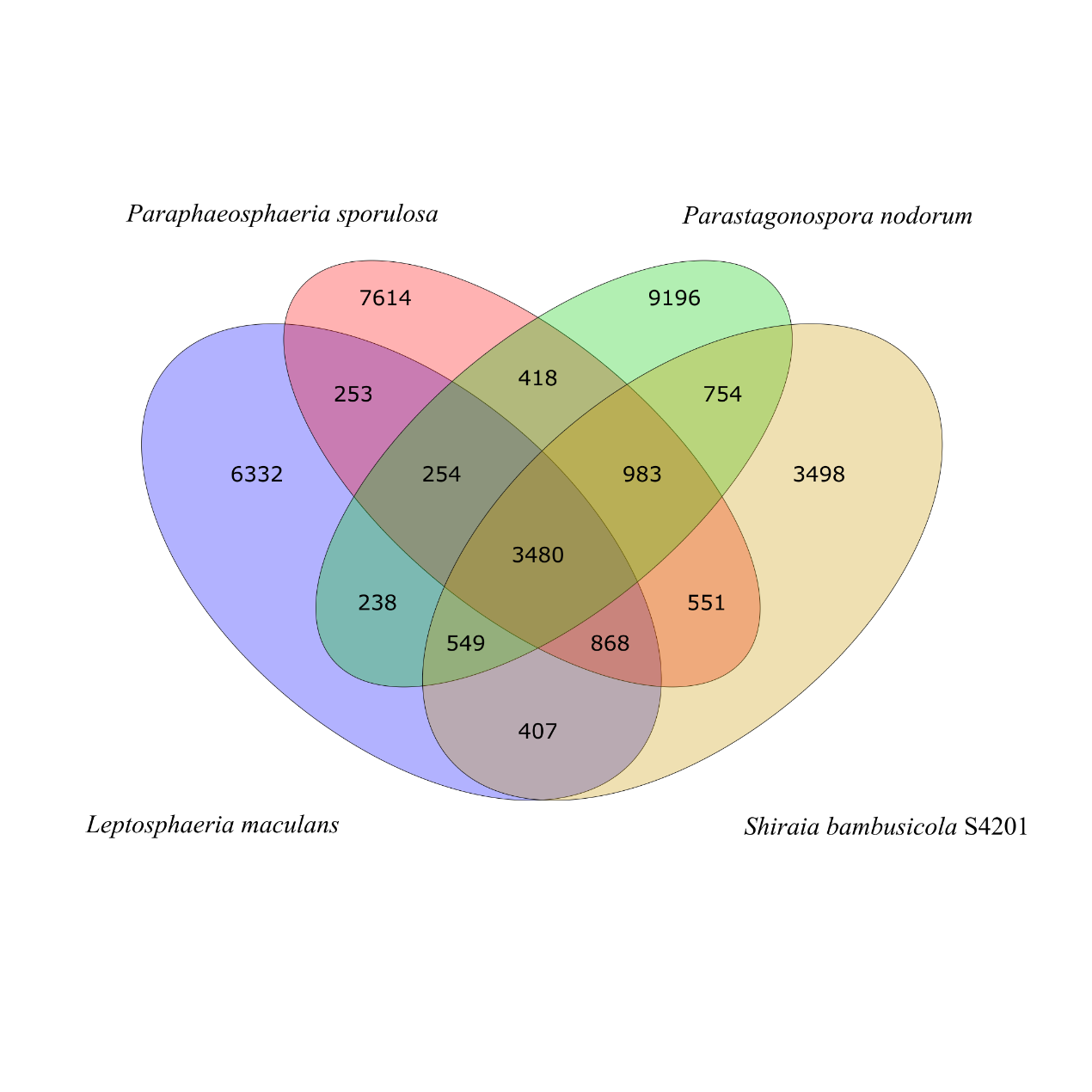


**Figure S7.** Comparative core and pan gene analysis of the four genomes.


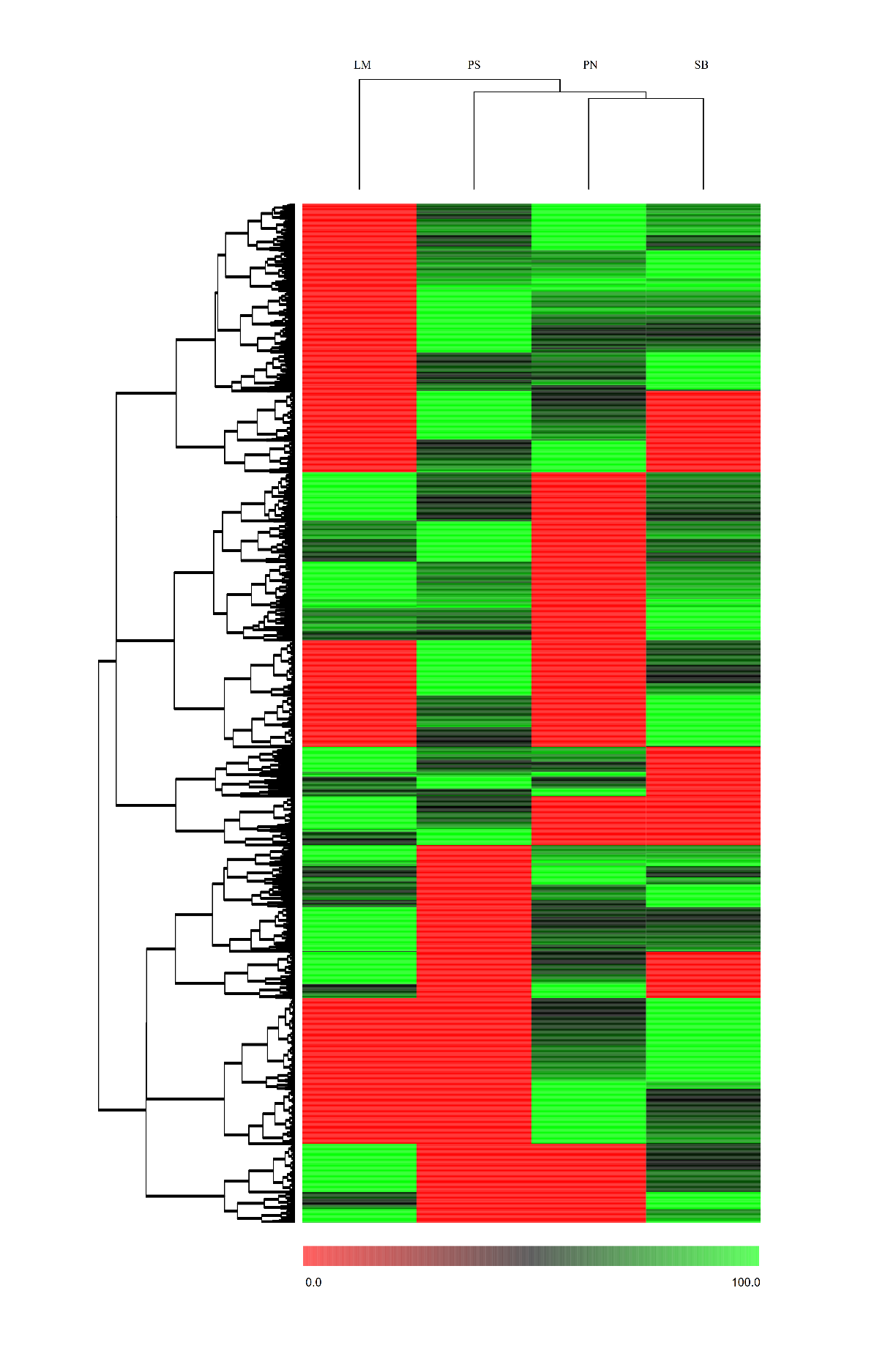


**Figure S8.** Hierarchical clustering of dispensable genes in four fungal species. The color key indicates gene homology from lowest (red) to highest (green). LM: *Leptosphaeria maculans*, PS: *Paraphaeosphaeria sporulosa*, PN: *Parastagonospora nodorum*, SB: *Shiraia bambusicola* S4201.


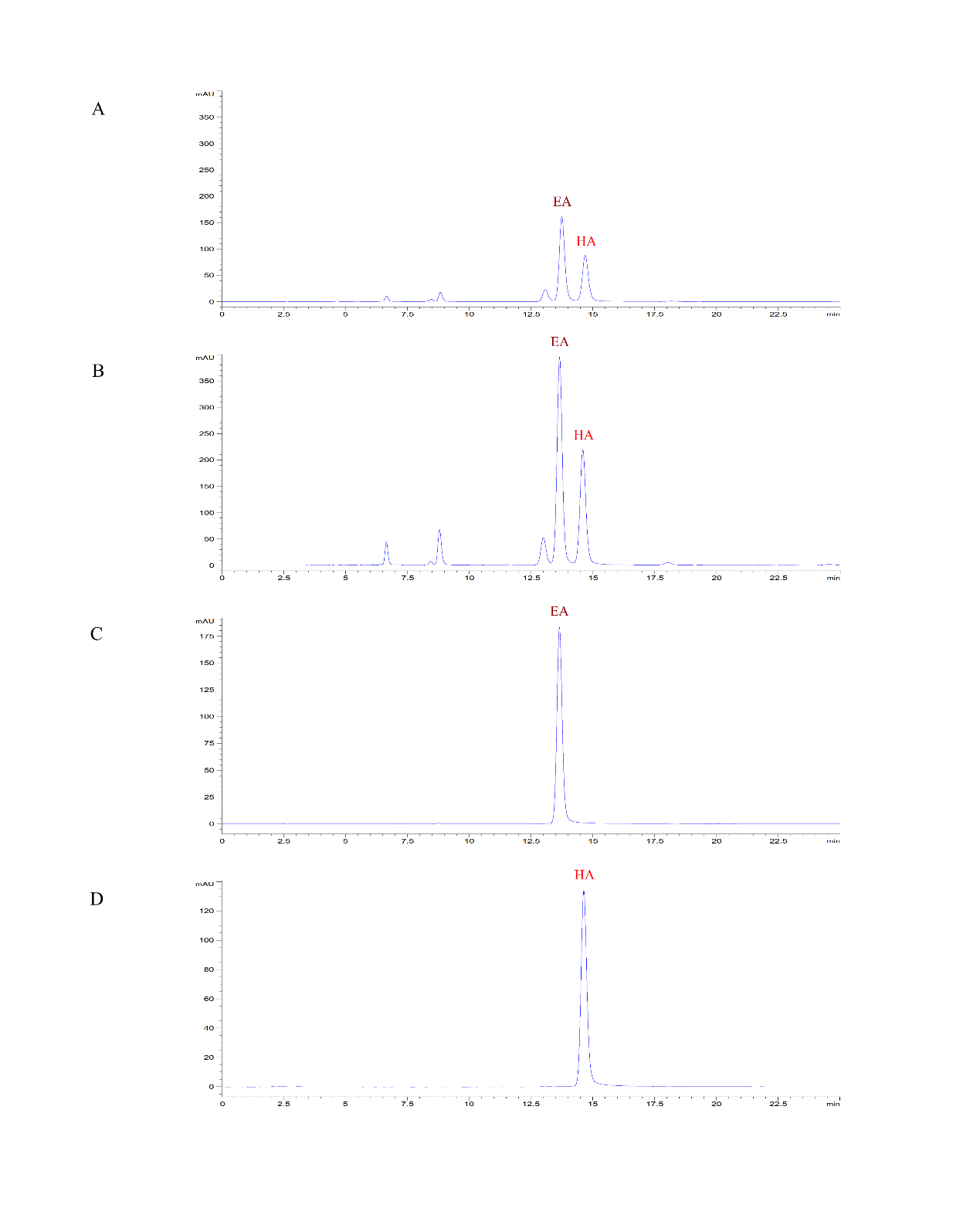


**Figure S9.** HPLC profiles (UV 460 nm) of culture extracts from *Shiraia bambusicola* S4201 and the overexpression transformant. (A) Pigments produced by S4201-W, (B) Pigments produced by OE-*zftf*, (C) EA standard, (D) HA standard.
